# Are Growth Performance and Fecal Score in Weaning Pigs Affected by the Inclusion Level of Potato Protein Concentrate and the Enclosed Glycoalkaloids in Iso-Nitrogenous Diets?

**DOI:** 10.3390/ani13213350

**Published:** 2023-10-27

**Authors:** Annette Lykke Voergaard, Leonardo Victor de Knegt, Johannes Gulmann Madsen, Jacob Dall

**Affiliations:** 1 KMC A.m.b.a., Herningvej 60, 7330 Brande, Denmark; 2 Department of Veterinary and Animal Sciences, University of Copenhagen, Grønnegårdsvej 2, 1870 Frederiksberg, Denmark; 3 Vilofoss A/S, Ballesvej 2, Snoghøj, 7000 Fredericia, Denmark

**Keywords:** glycoalkaloids, α-solanine, α-chaconine, potato protein concentrate, piglet feed, piglet growth performance, fecal score

## Abstract

**Simple Summary:**

Potato protein concentrate (PPC) has been included in piglet diets for many decades because of its properties with respect to piglets’ protein requirements. The inclusion level was limited because of the content of toxic anti-nutritional factors, such as glycoalkaloids (GA). Glycoalkaloid content in PPC is reduced via resource-requiring processing, but if piglets tolerate GA in levels like in the standard PPC, energy-consuming processing can be reduced. The concern when increasing standard PPC levels is the potential negative effects of GA on growth performance. In the experiment, it was shown that piglets’ growth performance was not affected negatively, nor was the fecal score, when fed increasing levels of PPC and hence increasing GA. All diets were formulated to meet the national nutritional requirements for weaner pigs. Interestingly, one of the dietary treatments with high inclusion of standard PPC (and thus GA) displayed greater performance as compared with the group fed a diet without standard PPC. Therefore, it was concluded that standard PPC can be included with high inclusion rates in weaner diets without negatively affecting growth performance or fecal score and that the focus must be on the GA content of the diets rather than the inclusion rate of the standard PPC.

**Abstract:**

Glycoalkaloids (GA) are anti-nutritional factors in standard potato protein concentrate (PPC) fed to piglets. Increasing levels of standard PPC was expected to affect growth performance and fecal score negatively. Seven-hundred-and-twenty pigs (7–30 kg) were fed one of the following four diets within three feeding phases (days 0–13, 13–24, and 24–45): control (CTRL), PPC standard inclusion (PPC-S; 4%, 2%, and 0%), high PPC inclusion (PPC-H; 8%, 3.5%, and 2%), and extremely high PPC inclusion (PPC-EH; 12%, 5%, and 3.5%). During days 0–13, CTRL displayed no difference in growth performance compared with the three experimental groups (PPC-S, PPC-H, and PPC-EH). During days 13–24, PPC-H achieved greater (*p* < 0.001) average daily feed intake (ADFI) compared to CTRL. During days 24–45, no differences between groups were observed. For the overall experimental period (0–45 days), PPC-H displayed greater average daily gain (ADG) (*p* = 0.010) and ADFI (*p* = 0.024) compared to CTRL. The feed conversion ratio (FCR) remained unaffected between the groups for all experimental periods. Increasing levels of PPC and hence GA did not affect the probability of diarrhea. In conclusion, increased standard PPC and hence increased levels of GA in isonitrogenous diets did not negatively affect growth performance nor fecal score in piglets (7–30 kg).

## 1. Introduction

In the European Union, there has been an increased focus on including protein feed ingredients from locally grown crops at the expense of imported protein, mainly originating from soybeans, sunflowers, and rapeseeds [1]. There is currently a tendency towards partly replacing protein of soybean origin in animal feed and thereby reducing the import of soybean meal (SBM) [1]. Using imported SBM as a feed ingredient in Europe is associated with relatively large transportation and energy costs, which, together with land use change, contributes to the overall climate footprint of the compound feed and therefore pig produced. Furthermore, there is an increased political and societal pressure to utilize already arable land rather than expanding it by deforestation. For this reason, the EU plant protein strategy aims to encourage the production of local plant proteins in the EU in an economically and environmentally sustainable manner [2].

In Europe, there are a number of locally grown protein crops suitable as pig feed ingredients, such as field peas, fava beans, rapeseeds, and sunflowers [3]. Side streams from the production of food ingredients are another potential source of protein for feed applications [4]. Amongst these is potato protein concentrate (PPC) from potato starch production.

PPC has been widely accepted and used as an ingredient in pig diets for several decades, but only with limited inclusion level and mainly in pre-starter diets for weaned piglets. Compared with other processed protein ingredients used in pre-starter diets, the content of lysine and other essential amino acids is relatively high in PPC [5,6]. However, due to the content of glycoalkaloids (GA), mainly α-solanine and α-chaconine [7], it is (in Denmark) advised not to exceed inclusion levels above 5% or 200 ppm solanine [8]. In human nutrition, the EU legislation prescribed a maximum limit of GA below 150 mg/kg in the ingredient [9] or 1 mg GA per kg bodyweight per day [10], but for animal nutrition, there is no maximum limit established due to insufficient data on occurrence in feed and on potential adverse effects of GA in animals according to the scientific opinion of EFSA on the topic [10].

Glycoalkaloids are toxic substances, which can elicit several adverse effects in both humans and animals, as reviewed by Friedman [11]. Furthermore, GA are known to have a bitter taste, at least in humans [11]. Pastuszewska et al. [12] analyzed batches of PPC from three different production sites produced under standard industrial conditions and found the content of GA in PPC varied from 927 to 2632 ppm. Other studies [5,6] have investigated GA levels in diets for weaned piglets, where, respectively, 78.8 ppm GA and 154.5 ppm GA in two different diets for newly weaned piglets (4–6 kg of BW) showed no adverse effect on growth performance [5], just as 118.5 ppm of GA in diets for 15 kg pigs did not elicit any adverse effect on growth or feed conversion [6]. The studies substituted different protein sources such as casein [6], soybean meal (SBM) [5,13], animal plasma [5], fish meal, and corn gluten meal [13] with PPC. However, these studies did not balance the digestible protein content nor the digestible amino acids content between the experimental diets, and therefore could not conclude whether the effect on growth performance was due to differences in digestible protein and amino acids or due to different levels of GA. In this regard, it is also important to note that inclusion levels of PPC in the diets varied greatly between the studies, from 3 to 5% [5] to 3.5–6% [13] to as high as 12.4 and 17.5% PPC [6].

In the standard extraction process of PPC for feed ingredient application, the GA content is reduced substantially. By additional processing steps, the GA content is reduced even further to reach the legislative maximum of GA for food ingredient application. These additional steps increase the amount of energy used in entire manufacturing [14]. Thus, if pigs can cope with the content of GA in the standard PPC or higher, it would greatly benefit the life cycle analysis of the PPC.

The main objective of the present study was to investigate the effect of increasing the amount of standard PPC to weaner pig isonitrogenous diets from 7 to 30 kg BW while substituting the protein ingredients; soy protein concentrate (SPC) (pre-starter, starter, and grower phase), fish meal (starter phase), and dehulled soy bean meal (grower phase) to determine the maximum tolerated level of α-solanine and α-chaconine from PPC and the effect on growth, feed intake, feed conversion ratio (FCR), and fecal score. It was hypothesized that increasing the levels of standard PPC in isonitrogenous pig diets from 0 to 45 days after weaning would affect growth performance negatively because of increasing GA content when substituting other protein ingredients, in this case, mainly SPC.

## 2. Materials and Methods

### 2.1. Animals, Treatments and Experimental Conditions

The experiment was conducted at the Livestock Feed Tests, Denmark, from April to June 2021. The herd functions as a contract research organization practicing relatively high standards concerning piglets, diet allocation, and common registrations (e.g., feed consumption, weight gain, and medical treatment) compared with standard commercial operations.

Seven hundred and twenty pigs [Duroc × (Landrace × Yorkshire)] with an average bodyweight (BW) of 7.04 kg ± SD 1.27 at weaning (day 0) were included in the experiment. Piglets were weaned at the age of approximately 28 days. Eighty pens were included in the experiment where, respectively, 40 pens housed 4 castrates and 5 female piglets and 40 pens housed 5 male and 4 female piglets. Thus, each pen housed 9 piglets of mixed sex. The initial BW was balanced between pens. All piglets included in the experiment were considered to have a high health status. During the entire experimental period, the piglets’ health status was monitored on a daily basis, and piglets displaying severe health issues (lameness, off-feed for 3 consecutive days, or respiratory problems) were excluded from the experiment and the growth performance analysis. Piglets were treated medically if needed and continued to be included in the experiment. Medical data were collected.

Each pen was equipped with a simple feeding trough with two spaces for eating separated by a trough divider. Drinking cups were placed on the outside of the feeding trough. Feed and water were provided ad libitum during the entire 45-day experimental period.

### 2.2. Diets and Feeding

Piglets were randomly allocated between four dietary treatments: a control diet (CTRL), a diet with a standard inclusion level of potato protein concentrate (PPC-S), a diet with a high inclusion level of potato protein concentrate (PPC-H), and a diet with an extremely high level of potato protein concentrate (PPC-EH). The diet compositions, including inclusion levels of potato protein concentrate (PPC), are shown in Table 1. Three feeding phases were applied in the 45-day experimental period: day 0 to 13 (pre-starter), day 13 to 24 (starter), and day 24 to 45 (grower).

All diets within each phase were formulated to be iso-energetic, as well as iso-nitrogenous, on a digestible basis. In addition, the amount of digestible essential amino acids (lysine, methionine, cysteine, threonine, tryptophane, and histidine) was balanced between the diets within each phase. Furthermore, digestible phosphorus was also balanced between the diets within each phase. All feed recipes met the nutrient requirements as recommended by SEGES Innovation [15].

Across the four diets from CTRL to PPC-EH, soy protein concentrate (SPC) was gradually substituted with PPC. To meet the nutrient requirements, the additional feed ingredients were adjusted within each diet when necessary. These adjustments were, in general, kept to a minimum to reduce the influence on diet composition. In this regard, when included in the diets, the ingredients fishmeal, whey powder and -protein, and extruded soybeans were kept at a constant level across diets. In the CTRL pre-starter diet, 1% of low glycoalkaloid PPC (α-solanine: 42 ppm; α-chaconine: 77 ppm) was needed to fulfill the requirements of amino acids.

The pre-starter diets were provided as “crumbled” feed (crushed pellets), whereas starter and grower diets were processed as pellets. The feed was produced as complete feed by DLG, Denmark. Potato protein concentrate was produced and supplied by KMC, Denmark.

### 2.3. Recordings and Calculations

Body weight was recorded on days 0, 3, 13, 24, and 45 by weighing the piglets pen-wise. From BW (including the BW of piglets leaving the trial due to death or hospitalization), the average daily gain (ADG) was calculated per piglet based on the actual active feeding days. Feed intake per pen was recorded per feeding phase as feed allocated subtracted the remaining amount of feed in the feeder when changing to the following phase. The average daily feed intake (ADFI) was calculated per pen on days 0, 3, 13, 24, and day 45. Feed intake per piglet was calculated in kg feed intake divided by the number of feeding days and the number of piglets. Based on ADG and ADFI, FCR per pig was calculated as kg feed intake per kg body weight gain (BWG). ADG, ADFI, and FCR were calculated for each feeding phase and for the overall period.

The fecal score was recorded three times a week in each pen in every week of the experimental period. These recordings were carried out using a scale from 0 to 3 (0: normal feces, 1: soft feces, 2: mild diarrhea, 3: severe diarrhea) as conducted by Marquardt et al. [16] by the same two trained persons throughout the complete trial with no prior knowledge of the dietary treatment allotment.

Piglets removed from a pen were recorded either as dead or moved to a hospital pen. Corrections with respect to pigs per pen were made when a pig was removed.

### 2.4. Analysis of Diets

A compiled sample of the exact PPC applied for the trial was analyzed for α-solanine and α-chaconine (NMKL No. 159 based on [7]).

All feed ingredients included in the diets were all analyzed for chemical composition according to the internal analysis program of DLG. The four diets were formulated based on the internal analysis of chemical composition.

### 2.5. Statistical Analysis

Data were analyzed using R studio version 4.4.2. The analyzed outcome variables were BW, ADG, ADFI, FCR, and fecal consistency scoring. The types of models used for performance and fecal scoring were different and will therefore be described separately.

#### 2.5.1. Performance Parameters

In the models for BW, ADG, ADFI, and FCR, data were analyzed using linear mixed-effects models, with, respectively, dietary treatment as fixed and the section as a random effect. The starting weight of each phase (diet shift) was included as a covariable in the model analyzing BW, ADG, and FCR. The analysis of BW, ADG, ADFI, and FCR was performed with a pen as the experimental unit. All models were subjected to ANOVA to evaluate the overall systematic effect. The least square means from the models was extracted with the function lsmeans and are presented with the standard error of the mean (SEM). The *p*-value for differences of the LS-means were extracted with the function pairwise and used to evaluate differences between treatment groups. Differences were considered significant at *p* < 0.05. All model residuals were assessed and confirmed to be normally distributed.

#### 2.5.2. Fecal Scoring

As previously described, fecal consistency was evaluated according to the score. Due to the small number of events categorized as “3” and due to the fact that the break-off points between scores “0” and “1” were often subjective, and both indicating that no diarrhea was observed, it was considered more biologically and statistically robust to analyze this variable in a dichotomous state, where “0” and “1” were recoded as “0” meaning “no diarrhea” and “2” and “3” were recorded as “1” meaning “diarrhea” as also conducted by Marquardt et al. [16]. A generalized mixed-effects model with Treatment as a fixed effect and Section as a random effect was used to evaluate if the predicted probability of diarrhea was significantly different between pigs receiving the different dietary treatments within each phase. The least square means for the models were extracted with the function lsmeans and presented with the standard error of the mean (SEM). The *p*-value for differences of the LS-means were extracted with the function pairwise and used to evaluate differences between treatment groups. Generalized models produced logit outputs but were converted to the probability of having diarrhea per analyzed category using the formula *p* = exp(logit)/(1 + exp(logit)) to accommodate a simpler interpretation.

## 3. Results

### 3.1. Performance

Pigs in the CTRL group displayed no difference in BW, ADG, ADFI, or FCR compared with pigs in the PPC-S, PPC-H, or the PPC-EH group during the first period (0–13 days) of the experiment (Table 2). During the second period (13–24 days), pigs in the PPC-H group displayed greater ADFI *(p* < 0.001) compared with pigs in the CTRL group and PPC-EH group but did not differ from the PPC-S group (Table 2). In the same period (13–24 days), a difference was also observed in BW (*p* < 0.001) and ADG (*p* = 0.001), with pigs in the PPC-S group having significantly higher values than CTRL and PPC-EH for both parameters (Table 2). In the last period of the experiment (days 24–45), BW again differed significantly between groups (*p* < 0.001), but with no specific group standing out as higher or lower, suggesting that the difference was not likely related to the feed formulations, as investigated in this study. In the overall experimental period (0–45 days), pigs in the PPC-H group displayed greater (*p* = 0.010) ADG compared with pigs in the CTRL group (Table 2) but did not differ from the PPC-S and PPC-EH groups. The pigs in the PPC-H group also reached the greatest ADFI (*p* = 0.024) compared to pigs in the CTRL group (Table 2). In the overall experimental period (0–45 days), the pigs displayed no difference in FCR between groups (Table 2).

### 3.2. Fecal Score

The predicted probabilities per group (estimated as LS-means) and pairwise comparisons between treatments are presented in Table 3. Results are not presented for the third period, as only one observation was scored as having diarrhea (original score 2), meaning that it was not possible nor necessary to analyze these data, as there were no differences among treatments. For the sake of transparency, it should be reported that the one observation with a fecal score of 2 was from the CTRL group. Overall, lower probabilities of diarrhea (shown as LS-means) were observed in pigs in the experimental groups, PPC-S, PPC-H, and PPC-EH, compared to the CTRL group, but that difference was rarely significant. Although the LS-means probability observed for the CTRL and PPC-H group in the second period is the same, the value observed for the CTRL group was found to be statistically significant from the others, while for the PPC-H group, it was not. That is an artifact of the rounding after conversion from logit to probability, and the original values, where the CTRL group has a higher logit than the other groups, can be checked in Supplementary Table S1. During the first period, the PPC-EH group showed a statistically significant 20% reduction in diarrhea when compared to the CTRL group, both in the full model (*p* = 0.003) and the adjusted pairwise comparison (*p* = 0.01). The present study could not detect a statistically significant difference in the predicted probability of diarrhea when increasing levels of standard PPC, hence increasing the levels of GA in piglet diets.

### 3.3. Mortality and Medical Treatment

Data on mortality were recorded for all groups, but the design of the experiment did not allow statistical analysis. The mortality in the CTRL group was 3.3% of entered piglets, the PPC-S group was 0.7%, the PPC-H group was 1.3%, and the PPC-EH group was 3.3%. The antibiotic treatment was equally recorded but not designed for statistical analysis. The antibiotic treatment in the CTRL group was 1.3%, the PPC-S group was 0.7%, the PPC-H group was 1.3%, and the PPC-EH group was 2.0%.

## 4. Discussion

Previous studies have investigated the effect of including PPC in piglet diets on growth performance [5,6,13] by focusing on the GA levels but not accounting for diets being isonitrogenous between dietary treatments regarding the digestibility of protein and digestible amino acids.

In this study, it was hypothesized that increasing the levels of standard PPC in isonitrogenous pig diets from 0 to 45 days after weaning would negatively affect growth performance because of the increased dietary GA content when substituting other protein ingredients, in this case, mainly SPC. 

In the present study, increasing the inclusion levels of standard PPC in weaner pig diets did not negatively affect ADFI. The PPC-H diet, resulting in the overall highest ADFI, included 8% (0–13 days), 3.5% (13–24 days), and 2% (24–45 days) of standard PPC. All diets included flavoring ingredients in similar inclusion levels within each feeding phase (0.9% 0–13 days, 0.6% 13–24 days, and 0.3% 24–45 days), as commonly used in commercial weaner diets. It cannot be excluded that the flavoring ingredients might have partly masked the bitter off-flavor associated with GA [11] present in the standard PPC and therefore not negatively affected the feed intake. All diets within the same feeding phase contained similar levels of flavoring ingredients; in fact, the PPC-EH diet contained less flavoring relative to the PPC level compared with the remaining diets, which possibly resulted in less masking of bitter off-flavor in this diet. However, there was no difference in ADFI between the CTRL and PPC-EH groups, despite the latter diet that contained the highest amount of standard PPC (12% 0–13 days, 5% 13–24 days, and 3.5% 24–45 days) and therefore the highest level of GA. This is in part contradictory to the findings of Kerr et al. [5], who conducted a study where a medicated feed was applied, containing medical preparations against Salmonella, *Escheridia coli*, and swine dysentery for increased weight gain and improved feed efficiency [17,18]. It has been suggested that certain medicine comes with flavor as medicine tends to have a bitter off-flavor [19]. In the study by Kerr et al. [5], all piglets received the same dosage of medicine in the diets but found decreasing ADFI with increasing levels of standard PPC compared with animal-based protein ingredients fed to pigs from 7 days after weaning to 28 days [5]. This suggests that no flavoring ingredients were included in the feed, or that if flavoring ingredients were included, they were not sufficient in masking the bitter-off taste of GA stemming from PPC, or finally, that pigs preferred the taste of animal-based protein. When comparing low GA PPC with diets containing animal-based protein, Kerr et al. [5] found multiple effects depending on the source: decreased ADFI when compared to animal plasma and no effect when compared to dried skim milk. In a similar study by Sardi et al. [13], which compared PPC to fish meal, sunflower meal, and corn gluten meal-based diets fed for 21 days from weaning, a greater ADG, and improved FCR in pigs fed PPC compared with all the included diets were reported. ADFI was not reported by Sardi et al. [13], but if calculated from the reported ADG and FCR, the ADFI for PPC-fed pigs was greater than for the pigs on the remaining diets. The observations combined stress the necessity of expanding the experimental design to include more diets based on different protein ingredients when examining the effect of substitution. Furthermore, the results from the aforementioned studies [5,13] indicate that flavoring ingredients and the inherent flavor of the ingredient in question should be carefully considered, especially when examining ADFI and growth performance in general. In the present study, PPC mainly substituted SPC. SPC contains different varieties of ANFs, e.g., trypsin inhibitors (TI). The anti-nutritional effects of GA are related to adverse physiological effects on different organs when fed at extremely high levels [11,20] and off-flavor [11]. Piglets are indeed sensitive to bitter off-flavor [21], and thus, GA may have an indirect adverse effect on growth caused by possible decreased feed intake, while TI from SPC has a direct adverse effect on growth caused by decreased protein digestibility [20]. Trypsin inhibitors may also exhibit a bitter off-flavor, but likely not to the same extent as GA, as TI causes less of a decrease in pig’s feed acceptability than does GA [19], which leads to the expectation that the substitution of SPC with PPC would impair the overall flavor of the experimental diets. Nevertheless, there was no adverse effect on the ADFI with increasing PPC. In this context, PPC might be closer related to animal-based protein ingredients in terms of flavor characteristics, while a negative effect on protein digestibility mediated by TIs may not be a major concern when including PPC at relatively high levels. The PPC applied in the present study was analyzed for TI, displaying relatively low levels.

In the present study, increasing the inclusion levels of standard PPC to 8, 3.5, and 2% (PPC-H diets), respectively, within phases, increased the ADG over the entire experimental period (0–45 days). Within days 13–24, the PPC-S-fed pigs reached the highest ADG of all groups. The high ADFI of PPC-H fed pigs, together with the high ADG, did not result in a poorer FCR, as there were no significant differences with respect to this parameter. This suggests that piglets adapt well to the increasing inclusion levels of standard PPC and that the dietary protein from the standard PPC diets is utilized to the same degree as the dietary protein of the CTRL group. This is supported by the findings of Tusnio et al. [6], who observed that the apparent ileal digestibility (AID) of protein and amino acids of standard PPC (677 ppm GA) in piglets of 21 kg BW was 76.9% for protein and 83% and 88% for lysine and methionine, respectively. For SPC, Navarro and Stein [22] found AID for protein fed to weaning pigs to be 81.94%, and for lysine and methionine, they found AID to be 84.47% and 90.25%, respectively.

Tusnio et al. [6] found increasing weight of piglet liver when fed PPC (118.5 ppm GA in the diet) compared to casein. Changes in liver weight are believed to be caused by the hepatoxic effects of GA [23]. In the present experiment, organs were not examined. Therefore, we cannot conclude that the increased levels of standard PPC and thereby increased levels of GA did not affect the piglets’ organs. In the study of Tusnio et al. [6], they found decreased dry matter content of feces when feeding PPC compared with casein, which may indicate challenges in the digestive system [24]. Fecal dry matter content is one parameter to study the effects of unfavorable conditions in the intestines; another parameter is fecal scoring, as conducted by Marquardt et al. [16]. The findings in this study suggested that fecal scoring was not affected when increasing GA levels in pig diets up to 141.6 ppm *α*-solanine and 204.84 ppm *α*-chaconine, as there could not be detected a statistically significant difference among the groups in the predicted probability of diarrhea, although the PPC-EH group tended to have lower LS-means than the other experimental groups.

Kerr et al. [5] concluded that an 8% level of low GA PPC in pre-starter (0–14 days) diets would be the maximum inclusion in pig diets to avoid negative effects on performance. In that experiment, 8% low GA PPC corresponded to 12.48 ppm GA. Compared with this study, Tusnio et al. [6] included older pigs (initial BW 15 kg, fed for 21 experimental days) in their study of feeding standard PPC (677 ppm GA) at a very high inclusion level (17.5%) corresponding to 118.5 ppm GA in the diet. No negative effects on growth performance were registered in the study by Tusnio et al. [6]. Sardi et al. [13] also fed newly weaned pigs (7.5 kg of BW) PPC at a 6% inclusion level and 3.5% from 15– to 30 kg of BW, but the GA content of the PPC or the diets was not reported. Sardi et al. [13] found that pigs fed the diet containing PPC outperformed pigs fed fish meal, sunflower meal, and corn gluten meal with respect to ADG, but there was no difference in FCR between the dietary treatments. In the present study, the growth performance in phase 0–13 days did not differ between pigs fed diets with 0% standard PPC (CTRL) and up to 12% standard PPC corresponding to 346.44 ppm GA (141.6 ppm *α*-solanine; 204.84 ppm *α*-chaconine), which is a substantially higher GA level compared with previous studies. Thus, rather than focusing on the PPC inclusion level itself, emphasis should be put on the total GA dietary content when considering the effect of PPC as an ingredient on growth performance.

Future studies are needed to investigate the physiological mechanisms behind the pig’s abilities to adapt to the relatively high dietary GA levels, as shown in the present study. Knowledge of toxic levels and the degree of adverse effects it might elicit on vital organs would be of great interest, in addition to GA interaction with the intestinal microflora, suggested to disrupt the cell membrane in the gastrointestinal tract [20].

## 5. Conclusions

It was concluded that increasing levels of standard potato protein concentrate (up to 12%) and hence increasing levels of glycoalkaloids (up to 346.44 ppm) did not negatively affect growth performance nor fecal score in the first 13 days, nor for the remaining experimental period (13–45 days) when fed isonitrogenous diets from day 0 to 45 after weaning when mainly substituting SPC. On the contrary, average daily gain and average daily feed intake were greater when including PPC in high amounts, and therefore relatively high dietary GA concentrations as compared with the control group, which did not include standard PPC.

## Figures and Tables

**Table 1:** Ingredient and nutrient composition of the experimental diets (CTRL, no standard potato protein concentrate (PPC); PPC-S, standard level; PPC-H, high level; PPC-EH, extreme high level).

Items	CTRL			PPC-S			PPC-H			PPC-EH		
Days of Experiment	0–13	13–24	24–45	0–13	13–24	24–45	0–13	13–24	24–45	0–13	13–24	24–45
Ingredients, %												
Cereals ^1^	52.61	71.26	68.8	54.81	71.57	68.8	56.01	72.45	70.21	57.07	72.17	71.25
Soy protein concentrate	14.9	6.8	1.5	9.9	4.8	1.5	5.1	3.1	-	-	2.7	-
Protein ingredients ^2^	17.2	4	-	17.2	4	-	17.2	3	-	17.4	2	-
Soybean meal	-	9	22	-	9	22	-	9	20.3	-	9	18
Potato protein concentrate ^3^	-	-	-	4	2	-	8	3.5	2	12	5	3.5
Low-glycoalkaloid potato protein concentrate ^4^	1	-	-	-	-	-	-	-	-	-	-	-
Synthetic amino acids	1.67	1.52	1.55	1.41	1.38	1.55	1.11	1.28	1.38	0.89	1.17	1.20
Vitamin- and mineral premix	0.4	0.6	0.6	0.4	0.6	0.6	0.4	0.6	0.6	0.4	0.6	0.6
Fats/oils ^5^	2.3	2,2	1.4	2.1	2.1	1.4	2.0	2.2	1.3	2.0	2.3	1.2
Miscellaneous feed additives	- ^6^	- ^7^	- ^8^	- ^6^	- ^7^	- ^8^	- ^6^	- ^7^	- ^8^	- ^6^	- ^7^	- ^8^
Calculated analysis												
ME, MJ/kg	14.7	13.6	13.4	14.7	13.6	13.4	14.7	13.6	13.4	14.7	13.6	13.4
SID protein, g/kg	174	155	160	174	155	160	174	155	160	175	154	161
SID Lys, g/kg	13.2	11.7	12.0	13.3	11.8	12.0	13.2	11.8	12.0	13.2	11.8	12.0
SID Met, g/kg	4.6	3.9	3.9	4.6	4.0	3.9	4.6	4.0	3.9	4.5	3.9	3.9
SID Met + Cys, g/kg	7.1	6.4	6.5	7.2	6.4	6.5	7.2	6.4	6.5	7.2	6.4	6.5
SID Thr, g/kg	8.3	7.3	7.5	8.3	7.3	7.5	8.2	7.3	7.5	8.3	7.3	7.4
SID Trp, g/kg	3.1	2.5	2.5	3.1	2.5	2.5	3.1	2.5	2.5	3.1	2.5	2.5
SID Ile, g/kg	7.0	5.8	7.5	7.3	5.8	7.5	7.6	6.2	7.5	8.0	6.3	7.4
SID Leu, g/kg	12.6	10.6	7.0	13.2	10.9	7.0	14.0	11.3	7.0	14.9	11.5	7.0
SID His, g/kg	4.0	3.5	2.5	3.9	3.4	2.5	3.8	3.4	2.5	3.8	3.5	2.5
SID Val, g/kg	8.6	7.5	7.7	8.6	7.5	7.7	8.9	7.5	7.7	9.7	7.5	7.7
Digestible P, g/kg	4.2	3.8	3.5	4.2	3.8	3.5	4.2	3.8	3.5	4.2	3.8	3.5
α-solanine, ppm ^9^	0.42	-	-	47.2	23.6	-	94.4	41.3	23.6	141.6	59	41.3
α-chaconine, ppm ^9^	0.77	-	-	68.28	34.14	-	136.56	59.75	34.14	204.84	85.35	59.75

^1^ **CTRL 0–13** (%): wheat: 34.99; wheat expanded: 10.62; extruded maize: 5.00; oat: 2.00. **PPC-S 0–13** (%): wheat: 34.95; wheat expanded: 12.86; extruded maize: 5.00; oat: 2.00. **PPC-H 0–13** (%): wheat: 34.91; wheat expanded: 14.00; extruded maize: 5.00; oat: 2.10. **PPC-EH 0–13** (%): wheat: 34.96; wheat expanded: 14.61; extruded maize: 5.00; oat: 2.50. **CTRL 13–24** (%): wheat: 47.46; oat: 3.00; barley: 20.8. **PPC-S 13–24** (%): wheat: 48.57; oat: 3.00; barley: 20.00. **PPC-H 13–24** (%): wheat: 49.45; oat: 3.00; barley: 20.00. **PPC-EH 13–24** (%): wheat: 49.17; oat: 3.00; barley: 20.00. **CTRL 24–45** (%): wheat: 46.80; oat: 2.00; barley: 20.00. **PPC-S 24–45** (%): wheat: 46.80; oat: 2.00; barley: 20.00. **PPC-H 24–45** (%): wheat: 48.21; oat: 2.00; barley: 20.00. **PPC-EH 24–45** (%): wheat: 49.25; oat: 2.00; barley: 20.00. ^2^ **CTRL 0–13** (%): fish meal: 3.00; extruded soybeans: 4.00; whey powder: 9.20; whey protein: 1.00. **PPC-S 0–13** (%): fish meal: 3.00; extruded soybeans: 4.00; whey powder: 9.20; whey protein: 1.00. **PPC-H 0–13** (%): fish meal: 3.00; extruded soybeans: 4.00; whey powder: 9.20; whey protein: 1.00. **PPC-EH 0–13** (%): fish meal: 3.20; extruded soybeans: 4.00; whey powder: 9.20; whey protein: 1.00. **CTRL 13–24** (%): fish meal: 2.00; fish protein hydrolyzed: 2.00. **PPC-S 13–24** (%): fish meal: 2.00; fish protein hydrolyzed: 2.00. **PPC-H 13–24** (%): fish meal: 1.00; fish protein hydrolyzed: 1.00. **PPC-EH 13–24** (%): fish protein hydrolyzed: 2.00. ^3^ *α*-solanine content: 1180 ppm; *α*-chaconine content: 1707 ppm. ^4^ *α*-solanine content: 42 ppm; *α*-chaconine content: 77 ppm. ^5^ **CTRL 0–13** (%): lecithin: 2.3. **PPC-S 0–13** (%): lecithin: 2.1. **PPC-H 0–13** (%): lecithin: 2.0. **PPC-EH 0–13** (%): lecithin: 2.0. **CTRL 13–24** (%): palm oil: 1.1; soy oil: 1.1. **PPC-S 13–24** (%): palm oil: 1.1; soy oil: 1.0. **PPC-H 13–24** (%): palm oil: 1.1; soy oil: 1.1. **PPC-EH 13–24** (%): palm oil: 1.2; soy oil: 1.1. **CTRL 24–45** (%): palm oil: 0.9; soy oil: 0.5. **PPC-S 24–45** (%): palm oil: 0.9; soy oil: 0.5. **PPC-H 24–45** (%): palm oil: 0.6; soy oil: 0.7. **PPC-EH 24–45** (%): palm oil: 0.6; soy oil: 0.6. ^6^ Misc. additives and more/kg: benzoic acid: 0.5%; phytase: 0.4%; β-xylanase: 0.005% (phytase and β-xylanase from premix); flavorings: 0.9%. ^7^ Misc. additives and more/kg: benzoic acid: 0.5%; phytase: 0.08%; β-xylanase: 0.05%; flavorings: 0.6%. ^8^ Misc. additives and more/kg: benzoic acid: 0.5%; phytase: 0.06%; β-xylanase: 0.05%; flavorings: 0.3%. ^9^ originating from potato protein concentrate.

**Table 2:** Productive performance of post-weaning pigs with diets with different levels of potato protein concentrate ^1^.

		CTRL ^2^	PPC-S	PPC-H	PPC-EH	SEM	*p*-Value
No. of pens		20	20	20	20		
Pigs entered		180	180	180	180		
**Performance parameters ^3^**							
BW, kg	Day 0	7.04	7.04	7.04	7.04		ns
	Day 13	10.43	10.57	10.66	10.36	0.17	0.567
	Day 24	14.84 ^a^	15.91 ^b^	15.47 ^ab^	15.09 ^a^	0.30	<0.001
	Day 45 ^4^	29.64	29.28	30.11	29.89	0.38	<0.001
ADG, g/kg	Day 0–13	211	221	226	207	10.36	0.549
	Day 13–24	428 ^a^	528 ^b^	485 ^ab^	447 ^a^	29.76	0.001
	Day 24–45	702	690	733	727	20.15	0.081
	Day 0–45	463 ^a^	492 ^ab^	508 ^b^	480 ^ab^	9.50	0.010
ADFI, kg/day	Day 0–13	0.19	0.21	0.21	0.19	0.01	0.323
	Day 13–24	0.62 ^a^	0.67 ^b^	0.67 ^b^	0.61 ^a^	0.02	<0.001
	Day 24–45	1.07	1.05	1.08	1.08	0.02	0.232
	Day 0–45	0.65 ^a^	0.68 ^ab^	0.70 ^b^	0.66 ^ab^	0.01	0.024
FCR, kg/kg	Day 0–13	0.92	0.97	0.93	0.93	0.04	0.736
	Day 13–24	1.52	1.32	1.42	1.37	0.09	0.181
	Day 24–45	1.52	1.52	1.48	1.50	0.04	0.455
	Day 0–45	1.40	1.39	1.38	1.37	0.02	0.620

^a,b^ Means in the same row with different superscripts differ significantly (*p* < 0.05). ^1^ Results are presented as least squares means and pooled SEM of the four dietary treatments. ^2^ Control diet (CTRL); standard inclusion of potato protein concentrate (PPC-S); high inclusion level of potato protein concentrate (PPC-H); extremely high inclusion level of potato protein concentrate (PPC-EH). ^3^ BW: body weight; ADG: average daily gain; ADFI: average daily feed intake; FCR: feed conversion ratio. ^4^ The ANOVA test indicates that the means of BW between experimental groups are overall different, but there is no significant specific pairwise difference between any two experimental groups.

**Table 3:** Diarrhea probability compared to population baseline. Fecal scores “0” and “1” = no diarrhea, and fecal scores “2” and “3” = a degree of diarrhea [16]. Pairwise comparison, difference in diarrhea probability (DP).

Experimental Period	Experimental Group ^1^		Diarrhea Probability (DP)	SE	*p*-Value
0–13 days	CTRL		0.67	0.61	0.110
	PPC-S		0.57	0.61	0.112
	PPC-H		0.60	0.61	0.267
	PPC-EH		0.47	0.61	0.003
13–24 days	CTRL		0.07	0.64	<0.001
	PPC-S		0.07	0.65	0.709
	PPC-H		0.07	0.64	<0.001
	PPC-EH		0.04	0.66	0.172
	**Pairwise Comparison**		**Difference in DP**	**SE**	***p*** **-Value**
0–13 days	CTRL vs.	PPC-S	0.11	0.57	0.385
		PPC-H	0.07	0.57	0.683
		PPC-EH	0.20	0.57	0.015
	PPC-S vs.	PPC-H	−0.03	0.57	0.955
		PPC-EH	0.10	0.57	0.505
	PPC-H vs.	PPC-EH	0.13	0.57	0.220
	CTRL vs.	PPC-S	0.01	0.62	0.982
		PPC-H	0.00	0.62	1.000
13–24 days		PPC-EH	0.04	0.64	0.521
	PPC-S vs.	PPC-H	0.03	0.62	0.982
		PPC-EH	0.03	0.64	0.713
	PPC-H vs.	PPC-EH	0.04	0.64	0.521

^1^ Control diet (CTRL); standard inclusion of potato protein concentrate (PPC-S); high inclusion level of potato protein concentrate (PPC-H); extremely high inclusion level of potato protein concentrate (PPC-EH).

## Data Availability

The data presented in this study are available in the article. Raw data is unavailable due to privacy restrictions, but can be retrieved through personal request.

## Supplementary Materials

The following supporting information can be downloaded at https://www.mdpi.com/article/10.3390/ani13213350/s1. Table S1: LS-means for the logit probability of diarrhea and pairwise comparison between groups. Fecal score 0 and 1 = no diarrhea, fecal score 2 and 3 = diarrhea.
