# Supplementary material for: Are Growth Performance and Fecal Score in Weaning Pigs Affected by the Inclusion Level of Potato Protein Concentrate and the Enclosed Glycoalkaloids in Iso-Nitrogenous Diets?
Source: Animals (Basel). 2023 Oct 27;13(21):3350. doi: 10.3390/ani13213350 (PMC10648507; doi:10.3390/ani13213350)
Supplement: Supplementary file 1 [file animals-13-03350-s001.zip › animals-2580978-supplementary.pdf]

animals

MDPI

Supplementary Table S1. LS-means for the logit probability of diarrhea and pairwise comparison between groups. Fecal score 0 and 1 = no diarrhea, fecal score 2 and 3 = diarrhea.

Experimental period	Experimental Group		LS-means (logit)	SEM	*p*-value
0-13 days	CTRL		0.71675	0.45	0.110
	PPC-S		0.26343	0.45	0.112
	PPC-H		0.40805	0.45	0.267
	PPC-EH		-0.12314	0.45	0.003
13-24 days	CTRL		-2.48723	0.67	<0.001
	PPC-S		-2.66377	0.67	0.709
	PPC-H		-2.48725	0.67	<0.001
	PPC-EH		-3.26128	0.67	0.172
	**Pairwise comparison**		**Difference in LS-means**	**SEM**	***p*-value**
0-13 days	CTRL vs.	PPC-S	0.45332	0.29	0.385
		PPC-H	0.30870	0.28	0.683
		PPC-EH	0.83989	0.28	0.015
	PPC-S vs.	PPC-H	-0.14462	0.28	0.955
		PPC-EH	0.38657	0.28	0.505
	PPC-H vs.	PPC-EH	0.53119	0.28	0.220
13-24 days	CTRL vs.	PPC-S	0.17654	0.47	0.982
		PPC-H	0.00002	0.48	1.000
		PPC-EH	0.77495	0.57	0.521
	PPC-S vs.	PPC-H	-0.17652	0.47	0.982
		PPC-EH	0.59841	0.56	0.713
	PPC-H vs.	PPC-EH	0.77493	0.57	0.521
